# Rapamycin-encapsulated nanoparticle delivery in polycystic kidney disease mice

**DOI:** 10.1038/s41598-024-65830-7

**Published:** 2024-07-02

**Authors:** Shinobu Yamaguchi, Randee Sedaka, Chintan Kapadia, Jifeng Huang, Jung-Shan Hsu, Taylor F. Berryhill, Landon Wilson, Stephen Barnes, Caleb Lovelady, Yasin Oduk, Ryan M. Williams, Edgar A. Jaimes, Daniel A. Heller, Takamitsu Saigusa

**Affiliations:** 1 Division of Nephrology, Department of Medicine, Section of Cardio-Renal Physiology and Medicine, McCallum Basic Health Science Building, University of Alabama at Birmingham, Room 533, 1918 University Blvd, Birmingham, AL 35233 USA; 2 Goldilocks Therapeutics, Inc., Bedford, NY USA; 3 Targeted Metabolomics and Proteomics Laboratory, University of Alabama at Birmingham, Birmingham, AL USA; 4 NanomediGene LLC, Birmingham, AL USA; 5 Department of Biomedical Engineering, The City College of New York, New York, NY USA; 6 Department of Medicine, Renal Service, Memorial Sloan Kettering Cancer Center, New York, NY USA; 7 Molecular Pharmacology Program, Memorial Sloan Kettering Cancer Center, New York, NY USA

**Keywords:** Medical research, Nephrology, Drug delivery, Nanomedicine

## Abstract

Rapamycin slows cystogenesis in murine models of polycystic kidney disease (PKD) but failed in clinical trials, potentially due to insufficient drug dosing. To improve drug efficiency without increasing dose, kidney-specific drug delivery may be used. Mesoscale nanoparticles (MNP) selectively target the proximal tubules in rodents. We explored whether MNPs can target cystic kidney tubules and whether rapamycin-encapsulated-MNPs (RapaMNPs) can slow cyst growth in *Pkd1* knockout (KO) mice. MNP was intravenously administered in adult *Pkd1*KO mice. Serum and organs were harvested after 8, 24, 48 or 72 h to measure MNP localization, mTOR levels, and rapamycin concentration. *Pkd1*KO mice were then injected bi-weekly for 6 weeks with RapaMNP, rapamycin, or vehicle to determine drug efficacy on kidney cyst growth. Single MNP injections lead to kidney-preferential accumulation over other organs, specifically in tubules and cysts. Likewise, one RapaMNP injection resulted in higher drug delivery to the kidney compared to the liver, and displayed sustained mTOR inhibition. Bi-weekly injections with RapaMNP, rapamycin or vehicle for 6 weeks resulted in inconsistent mTOR inhibition and little change in cyst index, however. MNPs serve as an effective short-term, kidney-specific delivery system, but long-term RapaMNP failed to slow cyst progression in *Pkd1*KO mice.

## Introduction

Autosomal dominant polycystic kidney disease (ADPKD) is the most common genetic kidney disorder, with over 50% of patients eventually developing end stage kidney disease. Most ADPKD patients carry mutations on the *PKD1* or *PKD2* genes, though kidney cysts appear earlier and progress more rapidly with *PKD1* mutations^1^. Currently, tolvaptan is the only Food and Drug Administration (FDA) approved drug to treat PKD^2,3^. However, tolvaptan results in polyuria and constant thirst. It also carries a risk for liver dysfunction, requiring patients to undergo routine bloodwork for surveillance. A well-known pathway that stimulates proliferation and cystogenesis in ADPKD is the mammalian target of rapamycin (mTOR) pathway^4,5^. Although mTOR inhibitors sirolimus and everolimus were shown to slow cyst growth in PKD rodents, they did not slow ADPKD in clinical trials^6,7^. This may be, in part, a result of dose reduction due to adverse effects, leading to inadequate kidney mTOR inhibition^8,9^. To overcome this limitation, and provide a safer, more effective dose, kidney-specific drug delivery could be utilized to circumvent drug metabolism by the liver.

Nanoparticles (NP), whose bio-distribution is dictated by surface material and size, have recently shown potential applications in diagnostics, imaging, and drug delivery, including cancer treatment^10^. FDA-approved poly lactic-*co*-glycolic acid (PLGA) polymers are physically strong, biocompatible, and biodegradable^11^. Unsurprisingly, these qualities make PLGA polymers an ideal surface material for drug-encapsulated NPs. To specifically target the kidney, particles must be large enough not to be quickly cleared from the body upon renal tubule bio-distribution (> 2 nm)^12,13^, yet small enough not to accumulate in the liver, spleen^14^, or lungs^15^ (< 100–1000 nm). Williams et al. have shown that PLGA conjugated to polyethylene glycol (PLGA-PEG) mesoscale nanoparticles (MNP), approximately 350 nm in size, selectively localize to mouse kidney proximal tubules more efficiently than to other organs^16^. Moreover, this MNP preferentially targets proximal versus distal tubules, identifying PLGA-PEG MNPs as potential kidney tubule-specific drug carriers^14,16^. In this study, we explored whether MNPs can target cystic kidney tubules and whether rapamycin-encapsulated MNPs can slow kidney cyst growth in *Pkd1* knockout (KO) mice.

## Results

### DEDC-MNP injection preferentially targets the kidney over other organs

**Figure 1:**
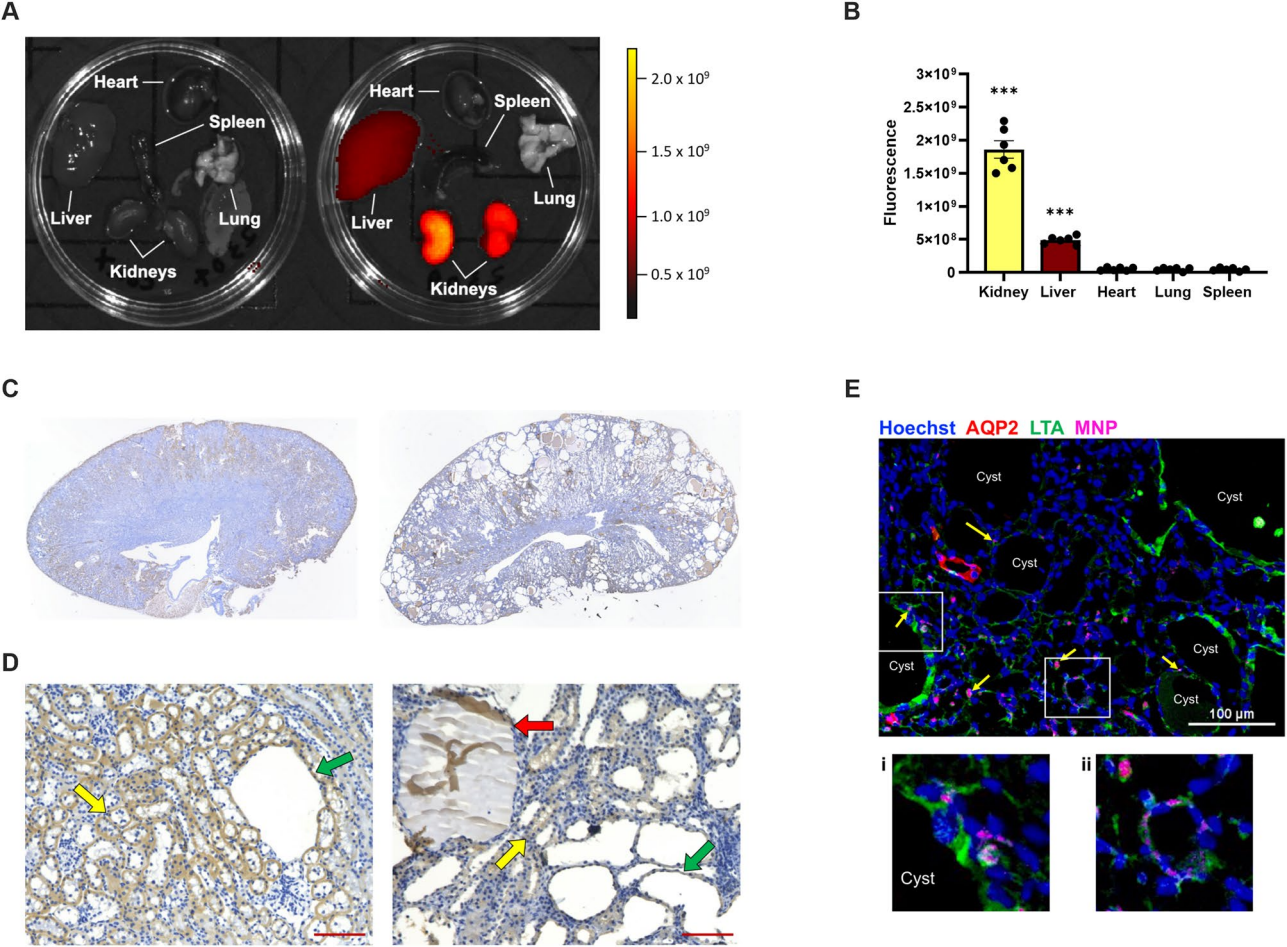
Intravenously injected DEDC-MNP localizes to the *Pkd1*KO mouse kidney. (**A**) Representative fluorescence image of various *Pkd1*KO mouse organs 24 h after a single intravenous (IV) injection of phosphate buffered saline (left; control) or DEDC-MNP (DEDC-conjugated mesoscale nanoparticles; 50 mg/kg) (right). The scale depicts highest (yellow) to lowest (black) fluorescent signal. (**B**) Kidneys, followed by liver, display the highest fluorescent signal post-IV injection of DEDC-MNP. (**C**) Representative images of polyethylene glycol (PEG) staining in mildly (left) and moderately (right) cystic kidneys at 4 × and (**D**) 10 × magnification (scale bar: 100 μm). Arrows point to non-cystic (yellow) and cystic (green) tubular epithelia, in addition to cystic lumen (red). (**E**) Representative immunofluorescent image of moderately cystic kidneys stained with Hoechst (nuclei, blue), aquaporin 2 (AQP2; distal tubule, red), and lotus tetragonolobus agglutinin (LTA; proximal tubule, green). DEDC-MNP (pink) localizes to (i) cystic (yellow arrows) and (ii) non-cystic proximal tubular epithelia (scale bar: 100 μm, 20 × magnification). Results of an Ordinary one-way ANOVA with Tukey’s multiple comparisons test reported. ***p < 0.001 compared to all other organs.

To determine the bio-distribution of MNP cargoes in vivo, DEDC-MNPs were injected into *Pkd1* KO mice and fluorescent intensity per organ was measured. Kidneys, followed by the liver, showed the highest intensity, with no observable signal in the heart, lung, or spleen (Fig. 1A,B). Since polyethylene glycol (PEG) is conjugated to the MNPs to prevent monocyte phagocytosis^17^, PEG staining was utilized to assess localization of the nanoparticles within the kidney. After a single injection, PEG was primarily found in the kidney cortex (Fig. 1C) of both mildly (left) and moderately (right) cystic kidneys. Moreover, PEG staining was observed in in both non-cystic (Fig. 1D; yellow arrow) and cystic (green arrow) tubular epithelia, with additional localization inside the cystic lumen (red arrow). Immunofluorescence imaging of moderately cystic kidneys likewise revealed MNP localization to both cystic (i) and dilated (ii) proximal tubular epithelia (Fig. 1E). Taken together, this suggests that intravenously injected MNPs accumulate in kidney epithelial cells, including cystic regions, as compared to other tissues.

### Encapsulated rapamycin elevates drug concentration and suppresses mTOR signaling

**Figure 2:**
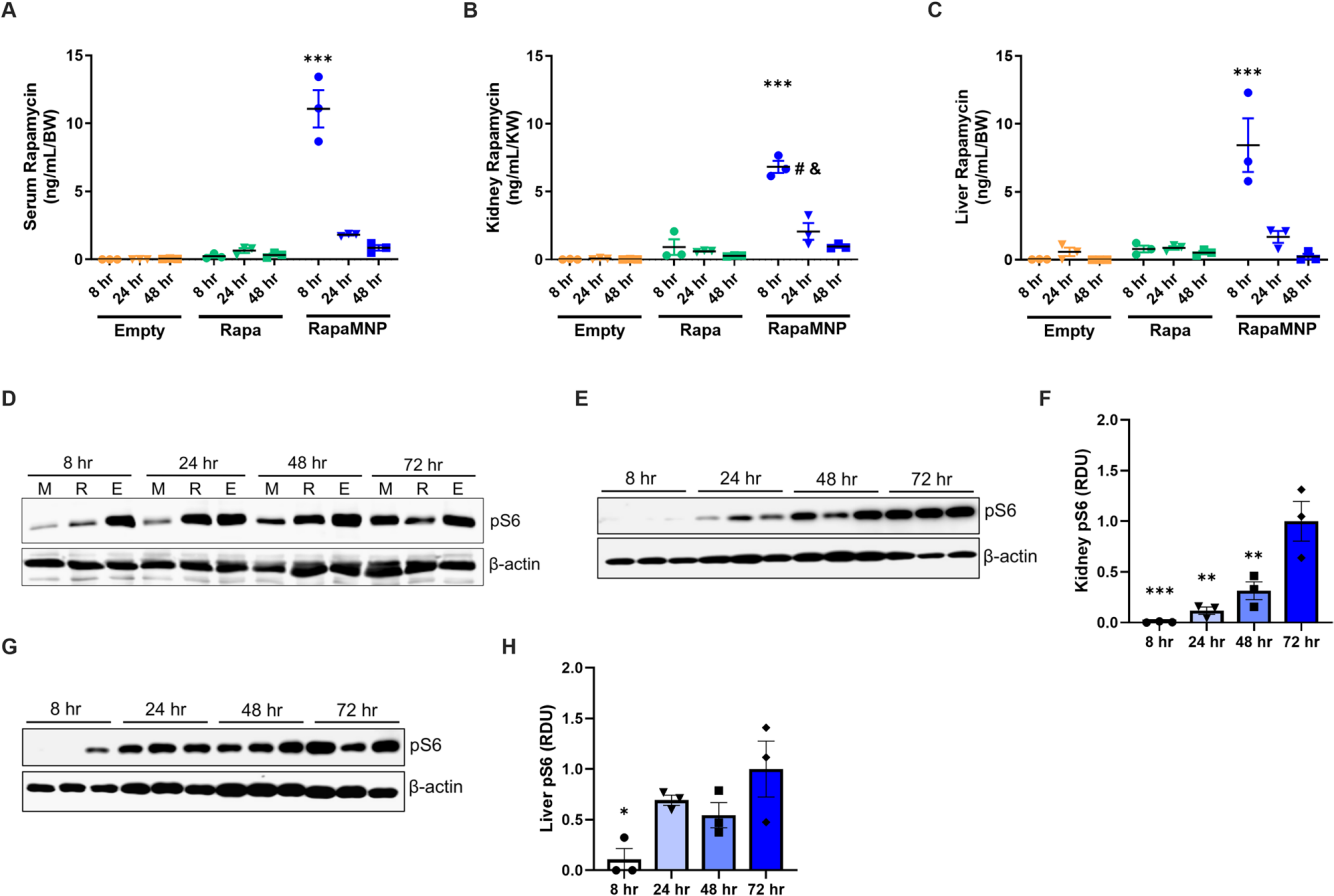
Rapamycin concentration and signaling after a single dose of empty MNP, free rapamycin (Rapa), or RapaMNP in *Pkd1*KO mice. (**A**) Serum, (**B**) kidney, and (**C**) liver rapamycin levels measured via mass spectrometry 8, 24, or 48 h after one IV injection (1 mg/kg) of respective compounds. Rapamycin concentration in all three locations was increased at 8 h by RapaMNP (normalized to body weight [BW] or kidney weight [KW]) compared to 24 and 48 h, as well as to the other treatments. In RapaMNP injected mice, kidney rapamycin concentration was still elevated after 24 h compared to other treatments at the same time. ***p < 0.001 compared to all other timepoints and treatment groups, ^#^*p* < 0.01 compared to other treatment groups at 24 h, ^&^*p* < 0.001 compared to same treatment at 8 h. (**D**) Representative Western blot of kidney phosphorylated S6 (pS6) timecourse in RapaMNP (M), Rapa (R), or empty MNP (E) dosed mice. Kidney pS6 is suppressed up to 48 h after a single dose of RapaMNP, but only 8 h after Rapa. (**E**, **F**) Representative Western blots and densitometric quantification of kidney and (**G**, **H**) liver pS6 post-IV RapaMNP injection. Phosphorylated S6 is suppressed in the kidney up to 48 h after a single dose, whereas the liver is only reduced at 8 h. Protein abundance is relative to 72 h. ***p < 0.001, **p < 0.01, *p < 0.05 compared to 72 h. Results of an Ordinary one-way or two-way ANOVA with Tukey’s multiple comparisons test reported. Full blot images in Supplement.

The pharmacokinetics and pharmacodynamics of encapsulated rapamycin (RapaMNP) were compared to empty nanoparticles and freely injected rapamycin (Rapa) in *Pkd1*KO mice after a single dose. Serum (Fig. 2A), kidney (Fig. 2B), and liver (Fig. 2C) rapamycin concentrations were highest in RapaMNP injected mice versus Rapa or empty MNP counterparts 8 h after injection. Kidney rapamycin levels remained elevated in RapaMNP injected mice until 24 h post-injection compared to other treatments. However, there were no significant differences in serum or liver rapamycin concentrations between empty MNP, Rapa, or RapaMNP after 24 or 48 h.

We next determined the duration of mTOR inhibition after a one-time injection of empty MNP, Rapa, or RapaMNP in the kidney by measuring phosphorylated S6 (pS6) levels. RapaMNP suppressed pS6 abundance for up to 48 h while free Rapa suppressed pS6 abundance for only 8 h (Fig. 2D). Protein abundance of pS6 was blunted up to 48 h post-injection in the kidneys (Fig. 2E,F), whereas pS6 was only reduced in the liver up to 8 h (Fig. 2G,H) after RapaMNP dosing. These results indicate that a single intravenous injection of RapaMNP results in higher drug delivery to the kidney compared to the liver and induces a sustained mTOR inhibition for approximately two days.

### Chronic RapaMNP treatment did not slow cyst growth in Pkd1KO mice

**Figure 3:**
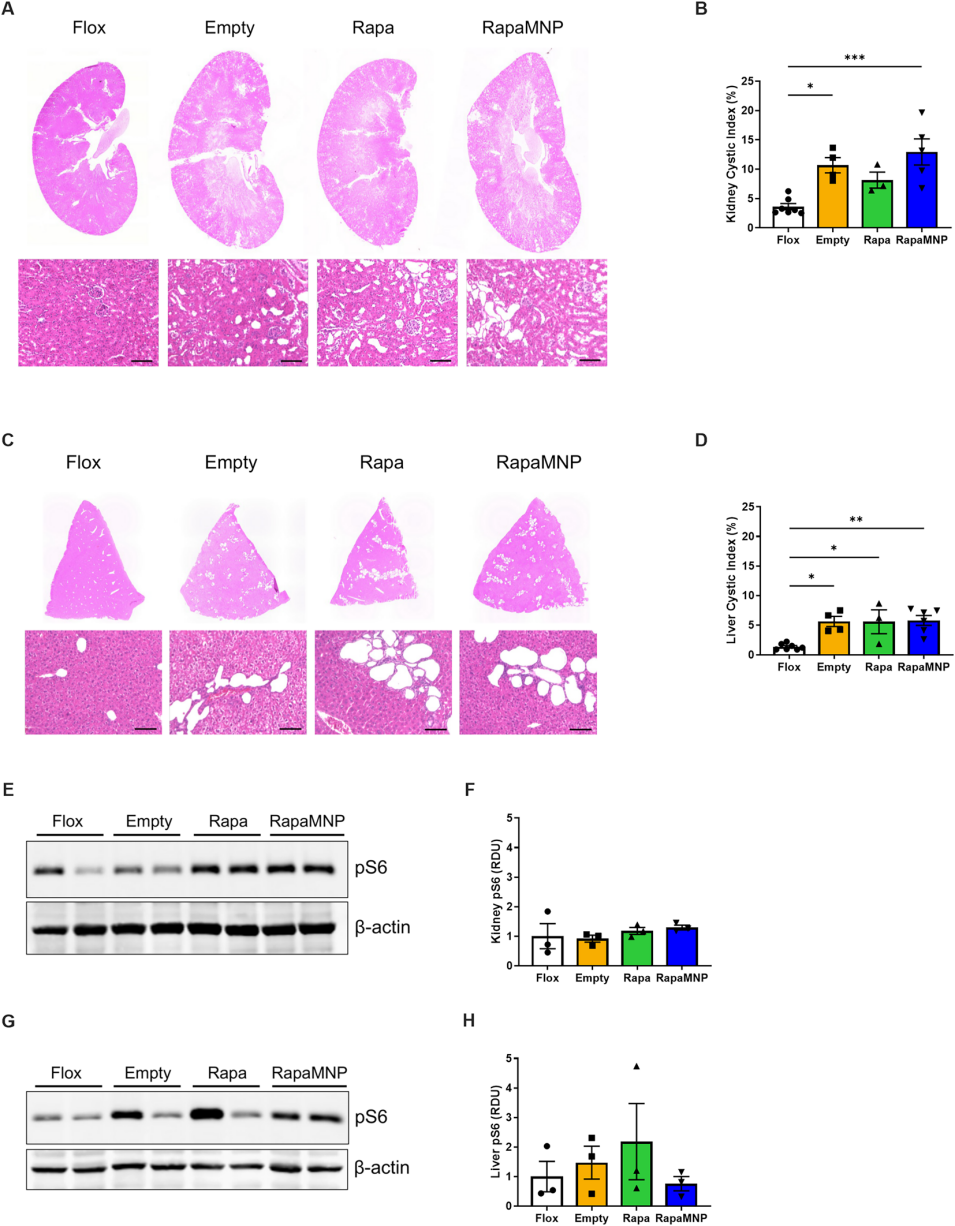
Chronic RapaMNP treatment does not improve cystic index or decrease pS6 abundance in *Pkd1*KO mice. (**A**, **B**) Representative kidney histology and cystic index. (**C**, **D**) Representative liver histology and cystic index. Top row: 4 × magnification whole tissue scan. Bottom row: 10 × magnification cyst image (scale bar: 100 μm). After 6 weeks of bi-weekly empty MNP, rapamycin, or RapaMNP IV injections (1 mg/kg), both kidney and liver cystic indexes were higher in *Pkd1*KO mice compared to flox mice, but not different amongst treatment groups. (**E**, **F**) Representative Western blots and densitometric quantification of kidney and (**G**, **H**) liver pS6 post-IV RapaMNP injection. No differences in pS6 abundance were observed between treatment groups in *Pkd1*KO mice. Results of an Ordinary one-way ANOVA with Tukey’s multiple comparisons test reported. ***p < 0.001, **p < 0.01, and *p < 0.05. Full blot images in Supplement.

Given its acute mTOR suppressive abilities, we further tested the long-term effect of RapaMNP in *Pkd1*KO mice. Ideally, the treatment schedule would be three times per week based on Fig. 2, however, due to technical difficulties with frequent tail vein injections, we instead treated *Pkd1*KO mice bi-weekly with either RapaMNP, rapamycin, or empty MNP for a total of 6 weeks. Despite an elevated cystic index in kidneys from *Pkd1*KO compared to flox mice (Fig. 3A,B), no reductions in the cystic index were observed after free or encapsulated rapamycin treatment. Similarly, liver cystic index was higher in *Pkd1*KO versus flox mice, but neither rapamycin nor RapaMNP treatment reduced cysts after 6 weeks of treatment (Fig. 3C,D). Inhibition of mTOR, as measured via pS6 abundance, was inconsistent in both kidney (Fig. 3E,F) and liver (Fig. 3G,H) tissue from *Pkd1*KO mice treated with RapaMNP compared to rapamycin or empty particles.

## Discussion

The aim of this study was to determine whether MNPs can target renal cystic epithelial cells and act as a feasible treatment option in ADPKD mice. Mesoscale particles (350 nm) were examined as their size limits liver fenestration entrance (100 nm) when administered intravenously^14^. We found that the kidney specificity of this particular NP in PKD mice was consistent with previous reports in control mice^14,16^. A single intravenous injection of fluorescent DEDC-MNPs resulted in higher uptake in the kidney, including cystic renal epithelia and surrounding interstitium, compared to the liver, heart, lung, or spleen. Furthermore, a single injection of rapamycin-encapsulated MNPs achieved higher and longer mTOR inhibition in the kidney versus liver of *Pkd1*KO mice. Despite these encouraging readouts, long-term treatment with rapamycin-encapsulated MNPs failed to slow cyst progression in our model of ADPKD.

Although we cannot be certain, we speculate that a major limitation of this study was the dosing regimen. Our data indicated that a single intravenous MNP injection increased circulating rapamycin concentration and mTOR inhibition in the kidney for approximately 48 h (Fig. 2B,D,E, and F). However, to mitigate tail damage due to frequent injection through tail vein, bi-weekly injections were instead performed over the 6 week period. In spite of these attempts, some mice still presented with stunted tails and vascular deformities by the end of the experiment. Therefore, although the NPs were injected without resistance, the shorter half-life and altered anatomical tail structure may have prevented the NPs from adequately entering circulation overtime. Shillingford et al. have shown that *Pkd1*KO mice respond to intraperitoneal rapamycin injections^18^, supporting the notion that rapamycin delivery, not the mouse model, acted as a limiting factor in the current study. In future strategies, perhaps an extended release formulation or longer-acting cargo, like mTOR small interfering RNA (siRNA), could help alleviate these difficulties.

Another item to consider when utilizing NPs is the feasibility of the drug delivery method in a disease that requires long-term treatment, as in ADPKD. Unlike chemotherapy, which can use a port/indwelling catheter for a short duration to treat cancer, PKD requires essentially lifelong treatment and frequent sterile intravenous treatment is unrealistic. Therefore, the ideal NP would be administered subcutaneously (SQ) or orally. Unfortunately, the MNP utilized in the present study was inadequate at achieving sufficient MNP levels in the blood, likely due to the large particle size^16^, when administered SQ or intraperitoneally (IP). Orally-administered, metformin-encapsulated NPs synthesized from chitosan were reported to be effective in PKD^19^, so this polymer material may be better suited for long-term treatment. The challenge with oral delivery, however, is to design a NP structure stable enough to tolerate the highly acidic gut environment but permeable enough to permit intestinal absorption into circulation without being compromised. If this oral NP indeed reaches the kidney successfully, it would become a candidate drug carrier for many kidney diseases.

All in all, this study indicates that MNPs serve as an effective short-term, kidney-specific delivery system. This is in agreement with the success of edaravone-loaded MNPs in preventing cisplatin-induced acute kidney injury^20^. Despite this success, life-long use, as is necessary with ADPKD, is not currently feasible given the method of delivery (Supplementary Figures).

## Methods

### PLGA-PEG construction

#### Modification of PLGA

Five milligrams of PLGA-COOH (Resomer RG504H 38,000–54,000 MW; 719,900-5G, Millipore Sigma, Burlington, MA) was weighed into a 20 mL scintillation vial. Separately, 135 mg of N-hydroxysuccinimide (NHS; 130,672-100 g, Millipore Sigma) and 230 mg of 1-ethyl-3-(3-dimethylaminopropyl)-carbodiimide (EDC; E6383-1G, Millipore Sigma) was added into a 1.5 mL tube. EDC and NHS were dissolved with PLGA-COOH in 10 mL methylene chloride (DCM; 270,997, Millipore Sigma) overnight on a stir plate. The polymer was precipitated in 10 mL of pre-chilled ethyl ether (E138-1, Fisher Scientific, Hampton, NH). Briefly, PLGA-NHS solution was added dropwise to cold ethyl ether, followed by three washes with 10 mL pre-chilled 1:1 methanol (A456-4, Fisher Scientific) to ethyl ether solution. The resulting polymer was vacuum dried for 4 h and stored at − 20 °C.

#### Conjugation of PEG to PLGA

One gram of PLGA-NHS was added to 250 mg NH2-PEG-COOH (PG2-AMCA-5 K, Nanocs Inc., New York, NY) and 37.7 uL of *N, N*-diisopropylethylamine (D125806, Millipore Sigma), then dissolved in 4 mL of chloroform (C606-4, Fisher Scientific). After overnight stirring reaction, PLGA-PEG was precipitated in 10 mL of pre-chilled methanol as mentioned above. The resulting polymer was vacuum dried and stored at − 20 °C.

### Nanoparticle preparation and characterization

Rapamycin (HY-10219, MedChem Express, Monmouth Junction, NJ) loaded PLGA-PEG MNPs were prepared using the nanoprecipitation method. Briefly, 100 mg of PLGA-PEG and 20 mg of rapamycin were dissolved in 2 mL of acetonitrile. This solution was added dropwise to a round bottom flask containing 4 mL of deionized water with 2% pluronic F-68 solution at a flow rate of 0.1 mL/min. After 2 h of stirring in a fume hood, MNPs were centrifuged at 7356 g in 50 mL conical tubes. MNPs were washed three times with 10 mL of water, resuspended in 10 mL of 5% sucrose solution, and lyophilized. The encapsulated amount of rapamycin was quantified using reversed-phase high-performance liquid chromatography (RP-HPLC). Briefly, 20 mg of MNPs were reconstituted in 1 mL of water, centrifuged at 33,000 g, then dissolved in 200 uL of acetonitrile. Rapamycin was quantified comparing absorbance against a standard curve. Average rapamycin loading was 6 µg per 1 mg of lyophilized MNPs. The release profiles of small molecules encapsulated in MNPs exhibit similar kinetics to each other and involve an intial burst release followed by a sustained release from the MNP^14,20^. We therefore predict that encapsulated rapamycin follows the initial burst release profile due to the compound’s hydrophobicity (theoretical log *P* value 4.63: source Pubchem). MNPs were also encapsulated with 3,3′-diethylthiadicarbocyanine iodide (Cy5) dye (DEDC) to fluorescently label. To do so, 10 mg of DEDC was dissolved with PLGA-PEG instead of rapamycin. The encapsulated amount of DEDC was quantified using UV–Vis spectrophotometry.

The physicochemical characterization of MNPs were measured by dynamic light scattering (DLS) using Malvern’s ZetaSizer Pro. Approximately 1 mg of lyophilized MNPs were reconstituted in 1X phosphate buffered saline (PBS) to measure hydrodynamic diameter and polydispersity index. Alternatively, MNPs were dissolved in water to measure zeta potential.

### Animals

Experimental protocols were approved by the Institutional Animal Care and Use Committee at the University of Alabama at Birmingham and performed in accordance with the National Institutes of Health Guide for the Care and Use of Laboratory Animals. Additionally, this study was reported in accordance with Animal Research: Reporting of In Vivo Experiments (ARRIVE) guidelines (https://arriveguidelines.org). Conditional *Pkd1* knockout (*Pkd1*KO) mice were generated by crossbreeding female *Pkd1*-floxed mice^21^ with male *Pkd1*-floxed mice containing a tamoxifen inducible systemic Cre (CAGG-CreER)^22^. Genotyping, performed using previously described primer sequences^21^, designated “flox” controls as mice lacking Cre. Male and female mice were equally utilized and maintained under constant temperature and humidity, a 12:12 h light–dark cycle, and water ad libitum. Flox and *Pkd1*KO mice (5–6 weeks old) received an intraperitoneal (IP) injection of Tamoxifen (T5648, Millipore Sigma; 9 mg/40 g) dissolved in corn oil every other day for a total of three doses. Kidney, liver, heart, lung, spleen, and serum were harvested under constant isoflurane inhalation followed by thoracotomy and either frozen at − 80 °C for biochemical analyses or immersed in respective buffers for histology or immunostaining.

### Nanoparticle delivery

Two weeks after tamoxifen induction, *Pkd1*KO mice were administered a single tail-vein injection (50 mg/kg, 200 μL) of mesoscale nanoparticles (MNP) encapsulating DEDC. The average particle size was 352.4 ± 9.6 nm with a polydispersity index (PDI) of 0.197 and zeta potential (ZP) of -39.2 mV. Subsequently, mice were dosed with either empty MNP (1 mg/kg), free rapamycin (Rapa) (1 mg/kg), or RapaMNP (1 mg/kg) to evaluate potency over time. The hydrodynamic diameter of RapaMNPs were 345.1 ± 5.0 nm with a 0.312 PDI and − 32 mV ZP. The loading of rapamycin was 6 µg per 1 mg of MNPs. In a separate study, *Pkd1*KO mice were chronically treated (1 mg/kg) via tail vein twice a week for a total of six weeks. Chronically treated mice were euthanized under constant isoflurane inhalation followed by thoracotomy one week after the final dose.

### Ex vivo fluorescent imaging

To measure tissue-specific delivery of administered nanoparticles, organs were harvested 24 h after a single tail vein injection (n = 3) and fluorescent intensity was captured using an IVIS 100 in vivo imaging system (PerkinElmer, Waltham, MA).

### Immunohistochemistry

Kidneys were fixed overnight in 10% formalin, followed by 70% ethanol until embedded in paraffin blocks. Embedded tissue was sectioned (5 μm), deparaffinized, and hydrated with decreasing amounts of ethanol. Following an incubation in 0.3% hydrogen peroxide solution, slides were heated in antigen retrieval buffer (00-4956-58, Invitrogen, Waltham, MA), then blocked for 30 min with 10% normal rabbit serum. Slides were incubated for 5 h at 4 °C with PEG primary antibody (ab94764, Abcam; 1:200) and 1 h at room temperature with biotinylated rabbit anti-rat secondary antibody (PK-4004, Vector Laboratories, Newark, CA; 1:200) prior to staining with 3,3′-diaminobenzidine (DAB; SK-4100, Vector Laboratories) and dehydration.

### Immunofluorescent Staining

Kidneys were stained as previously described^23^. Briefly, 30% sucrose cryopreserved tissue sections were fixed with 4% paraformaldehyde and permeabilized with 0.2% Triton X-100, then blocked with 2% donkey serum (ab7481, Abcam) before incubation with aquaporin 2 (AQP2) (ab62628, Abcam) primary and goat anti-rabbit IgG Alexa Fluor 647 (A21244, Invitrogen) secondary antibodies. Slides were subsequently stained with fluorescein isothiocyanate (FITC)-conjugated lotus tetragonolobus agglutinin (LTA) (FL-1321, Vector Laboratories) and Hoechst 33,342 nuclear stain (H3570, Fisher Scientific). Fluorescent images were captured using a Nikon TI2 Eclipse (Nikon Instruments) spinning disc confocal microscope equipped with a Yokogawa 31 disc (Yokogawa) on an Orca Flash 4.0 sCMOS (Hamamatsu) using a 403 Plan Fluor 1.3NA (Nikon Instruments) objective.

### Liquid chromatography tandem mass spectrometry

Rapamycin was quantified using liquid chromatography-tandem mass spectrometry (LC–MS/MS) with ascomycin (A-094, Cerilliant Corp., Round Rock, TX) serving as an internal standard. The standard curve was established with nine calibrants, offering a linear range of 0.1–1000 ng/mL. Serum and tissue samples were prepared by thawing on ice, spiking with ascomycin, and processing with Phree Phospholipid Removal (8BS133TAK, Phenomenex, Torrance, CA). Samples were then mixed with 1% formic acid in acetonitrile, filtered, dried under nitrogen gas, and reconstituted in 50% methanol. Tissue samples underwent an additional homogenization step using a bead beater homogenizer prior to filtering. The prepared samples and calibrants were analyzed using a Shimadzu Prominence 20 HPLC in tandem with a Sciex 6500 Qtrap Mass Spectrometer. The LC–MS/MS conditions were sourced from Phenomenex tech note TN-1169, ensuring the reliability and reproducibility of the analysis. This comprehensive process ensured the samples were adequately prepared for subsequent analysis.

### Western blot

Flox and *Pkd1KO* kidney tissue samples were homogenized and probed as previously described^24^. Membranes were blocked with 5% PhosphoBLOCKER Blocking Reagent (AKR-103, Cell Biolabs, Inc., San Diego, CA) for 30 min followed by a 4 °C overnight incubation with phospho-S6 ribosomal protein (Ser240/244; 5364, Cell Signaling, Danvers, MA) primary antibody and goat anti-rabbit IgG, DyLight 680 (35,569, Fisher Scientific) for 1 h at room temperature. Equal protein loading was verified by β-actin staining (3700, Cell Signaling; followed by goat anti-mouse IgG, DyLight 800 [SA5-10176], Fisher Scientific) and shown in each representative figure.

### Cyst quantification

Kidney and liver Sects. (5 μm) were cut and stained with hematoxylin–eosin (H&E). Whole kidney images were captured at 4× magnification using a Keyence BZ-X710 (Itasca, IL) microscope and analyzed using Image J as previously published^24^. Larger, representative cyst images were captures at 10× magnification.

### Statistical analyses

Data are presented as means ± SEM. Differences between group means were analyzed using GraphPad Prism 10 (La Jolla, CA) by Ordinary one-way or two-way ANOVA with Tukey’s multiple comparisons post hoc test, as noted. *P* < 0.05 denoted statistically significant.

### Supplementary Information

Supplementary Figures.

## Data Availability

The data generated and analysed in the current study are available from the corresponding author upon reasonable request.

## References

[1] Harris PC, Torres VE (2009). Polycystic kidney disease. Annu. Rev. Med..

[2] Torres VE (2012). Tolvaptan in patients with autosomal dominant polycystic kidney disease. N. Engl. J. Med..

[3] Torres VE (2017). Tolvaptan in later-stage autosomal dominant polycystic kidney disease. N. Engl. J. Med..

[4] Shillingford JM (2006). The mTOR pathway is regulated by polycystin-1, and its inhibition reverses renal cystogenesis in polycystic kidney disease. Proc. Natl. Acad. Sci. USA.

[5] Torres VE (2010). Prospects for mTOR inhibitor use in patients with polycystic kidney disease and hamartomatous diseases. Clin. J. Am. Soc. Nephrol..

[6] Serra AL (2010). Sirolimus and kidney growth in autosomal dominant polycystic kidney disease. N. Engl. J. Med..

[7] Walz G (2010). Everolimus in patients with autosomal dominant polycystic kidney disease. N. Engl. J. Med..

[8] Novalic Z (2012). Dose-dependent effects of sirolimus on mTOR signaling and polycystic kidney disease. J. Am. Soc. Nephrol..

[9] Canaud G (2010). Therapeutic mTOR inhibition in autosomal dominant polycystic kidney disease: What is the appropriate serum level?. Am. J. Transplant..

[10] Rezvantalab S (2018). PLGA-based nanoparticles in cancer treatment. Front. Pharmacol..

[11] Makadia HK, Siegel SJ (2011). Poly lactic-co-glycolic acid (PLGA) as biodegradable controlled drug delivery carrier. Polymers.

[12] Dolman ME, Harmsen S, Storm G, Hennink WE, Kok RJ (2010). Drug targeting to the kidney: Advances in the active targeting of therapeutics to proximal tubular cells. Adv. Drug Deliv. Rev..

[13] Zhou P, Sun X, Zhang Z (2014). Kidney-targeted drug delivery systems. Acta Pharm. Sin. B.

[14] Williams RM (2015). Mesoscale nanoparticles selectively target the renal proximal tubule epithelium. Nano Lett..

[15] Kim I (2012). Doxorubicin-loaded highly porous large PLGA microparticles as a sustained-release inhalation system for the treatment of metastatic lung cancer. Biomaterials.

[16] Williams RM (2018). Selective nanoparticle targeting of the renal tubules. Hypertension.

[17] Mathaes R, Winter G, Besheer A, Engert J (2014). Influence of particle geometry and PEGylation on phagocytosis of particulate carriers. Int. J. Pharm..

[18] Shillingford JM, Piontek KB, Germino GG, Weimbs T (2010). Rapamycin ameliorates PKD resulting from conditional inactivation of Pkd1. J. Am. Soc. Nephrol..

[19] Wang J (2021). Oral delivery of metformin by chitosan nanoparticles for polycystic kidney disease. J. Control Release.

[20] Williams RM (2021). Kidney-targeted redox scavenger therapy prevents cisplatin-induced acute kidney injury. Front. Pharmacol..

[21] Piontek KB (2004). A functional floxed allele of Pkd1 that can be conditionally inactivated in vivo. J. Am. Soc. Nephrol..

[22] Hayashi S, McMahon AP (2002). Efficient recombination in diverse tissues by a tamoxifen-inducible form of Cre: A tool for temporally regulated gene activation/inactivation in the mouse. Dev. Biol..

[23] Zimmerman KA (2020). Interferon regulatory factor-5 in resident macrophage promotes polycystic kidney disease. Kidney360.

[24] Sedaka R (2023). Accelerated cystogenesis by dietary protein load is dependent on, but not initiated by kidney macrophages. Front. Med..

