# Supplementary material for: Rapamycin-encapsulated nanoparticle delivery in polycystic kidney disease mice
Source: Sci Rep. 2024 Jul 2;14:15140. doi: 10.1038/s41598-024-65830-7 (PMC11219830; doi:10.1038/s41598-024-65830-7)
Supplement: Supplementary file 1 — Supplementary Figures. [file 41598_2024_65830_MOESM1_ESM.pdf]

Figure 2D

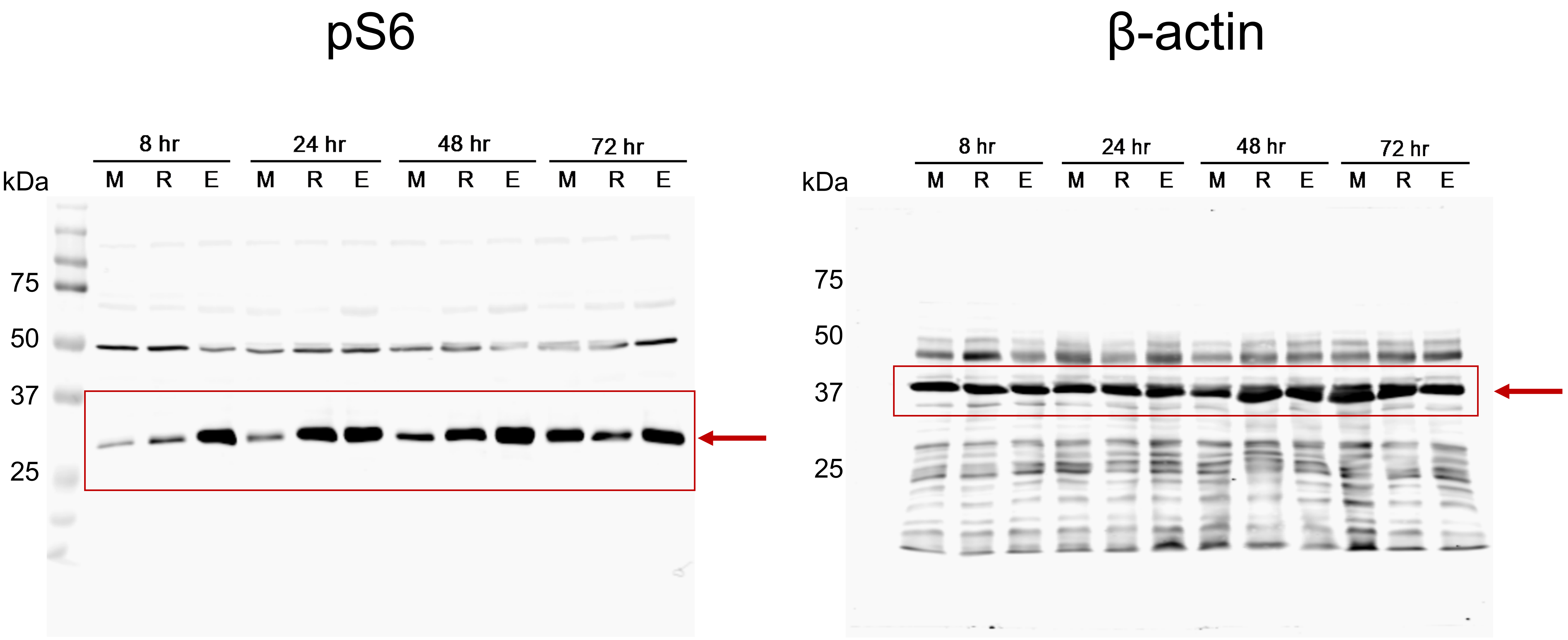

pS6
8 hr
24 hr
48 hr
72 hr
kDa
M R E M R E M R E M R E
75
50
37
25
β-actin
8 hr
24 hr
48 hr
72 hr
kDa
M R E M R E M R E M R E
75
50
37
25

Figure 2E

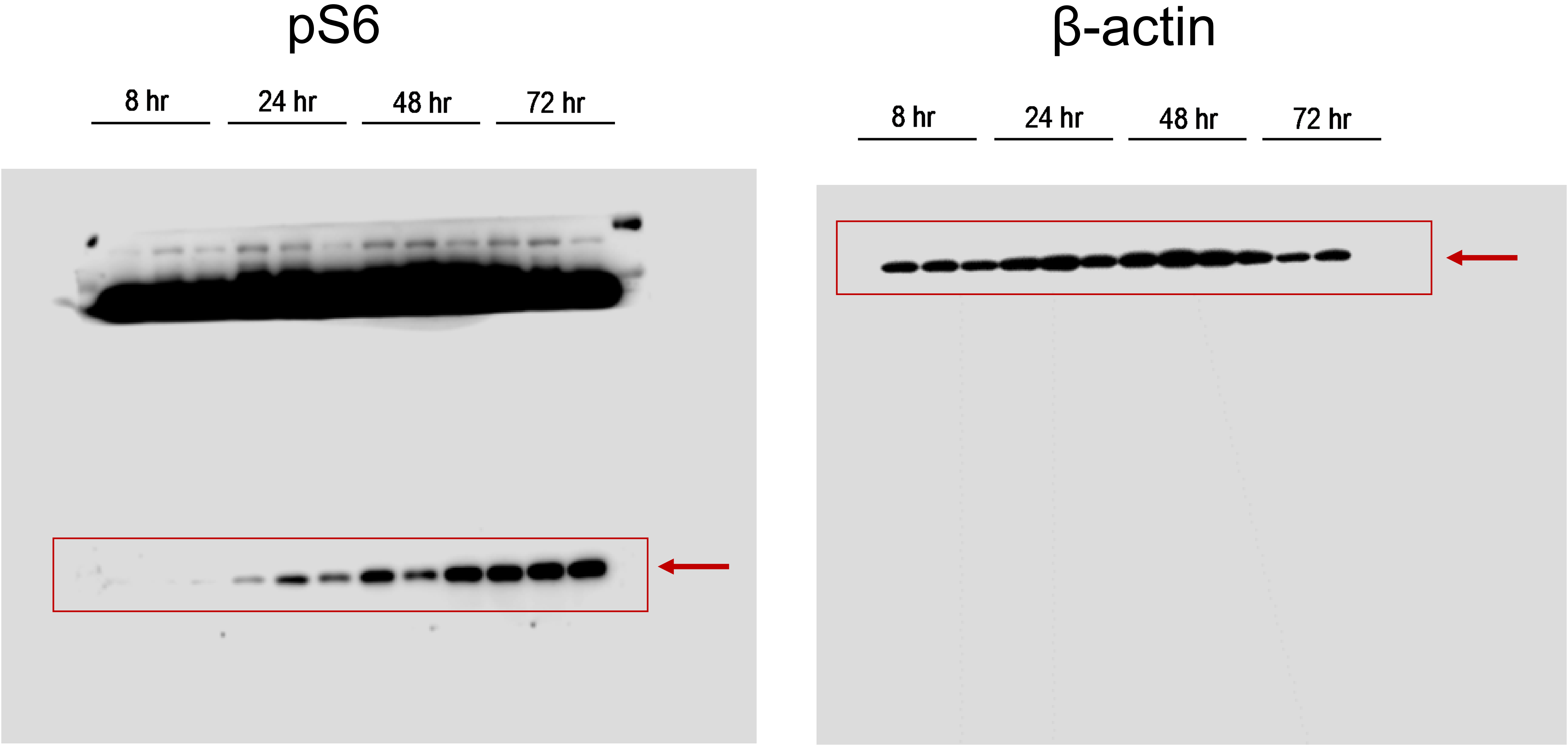

Figure 2G

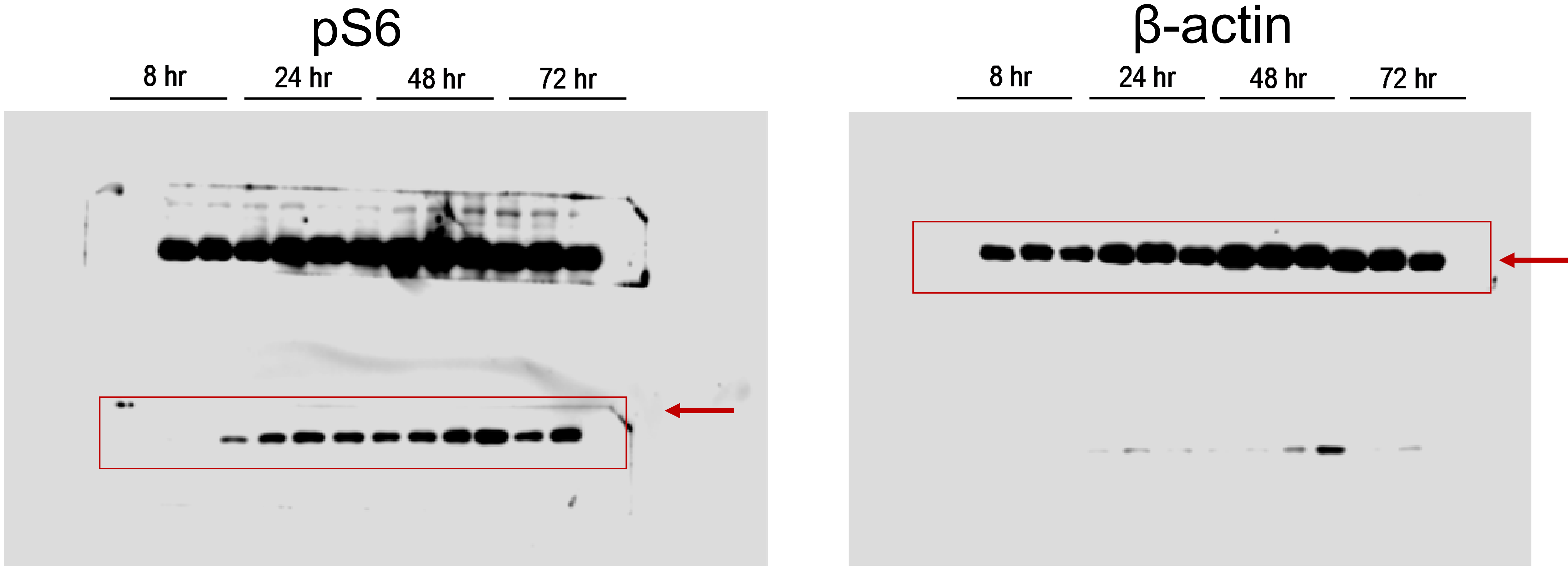

Figure 3E

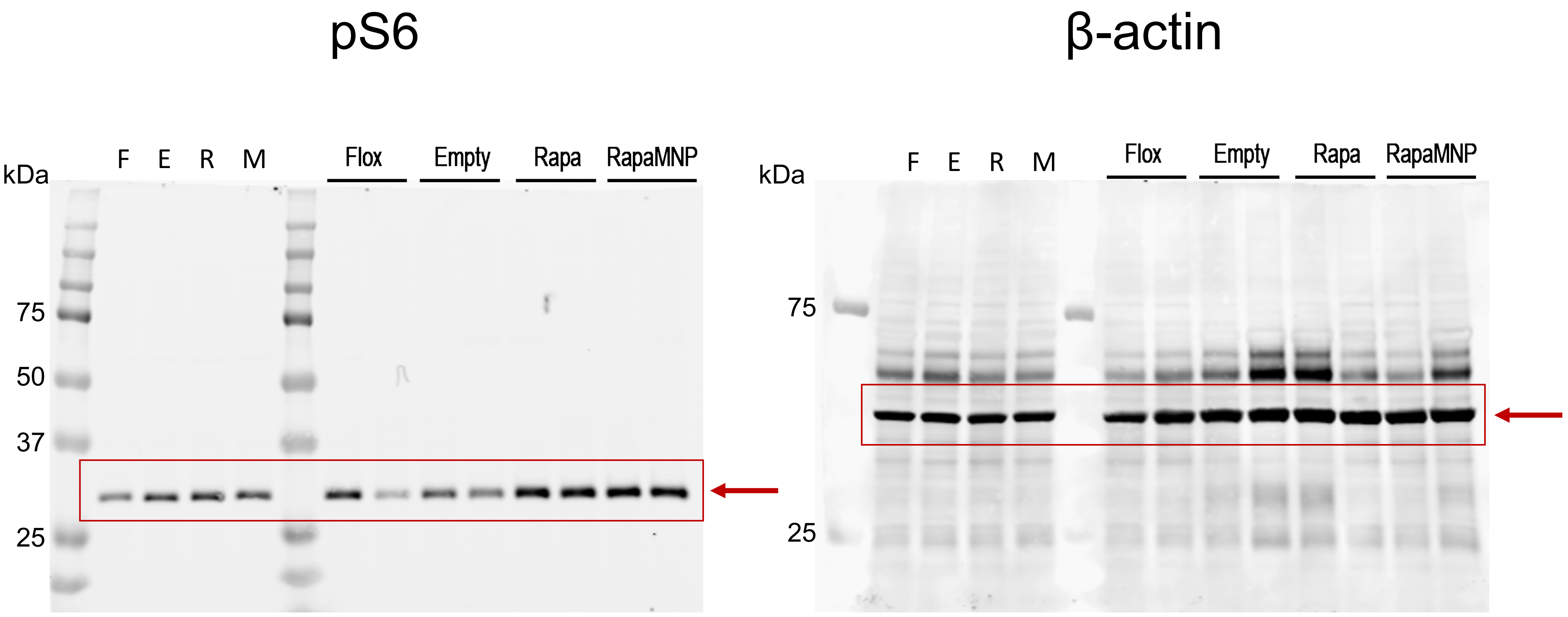

F- Flox E- Empty R-Rapa M- RapaMNP

Figure 3G

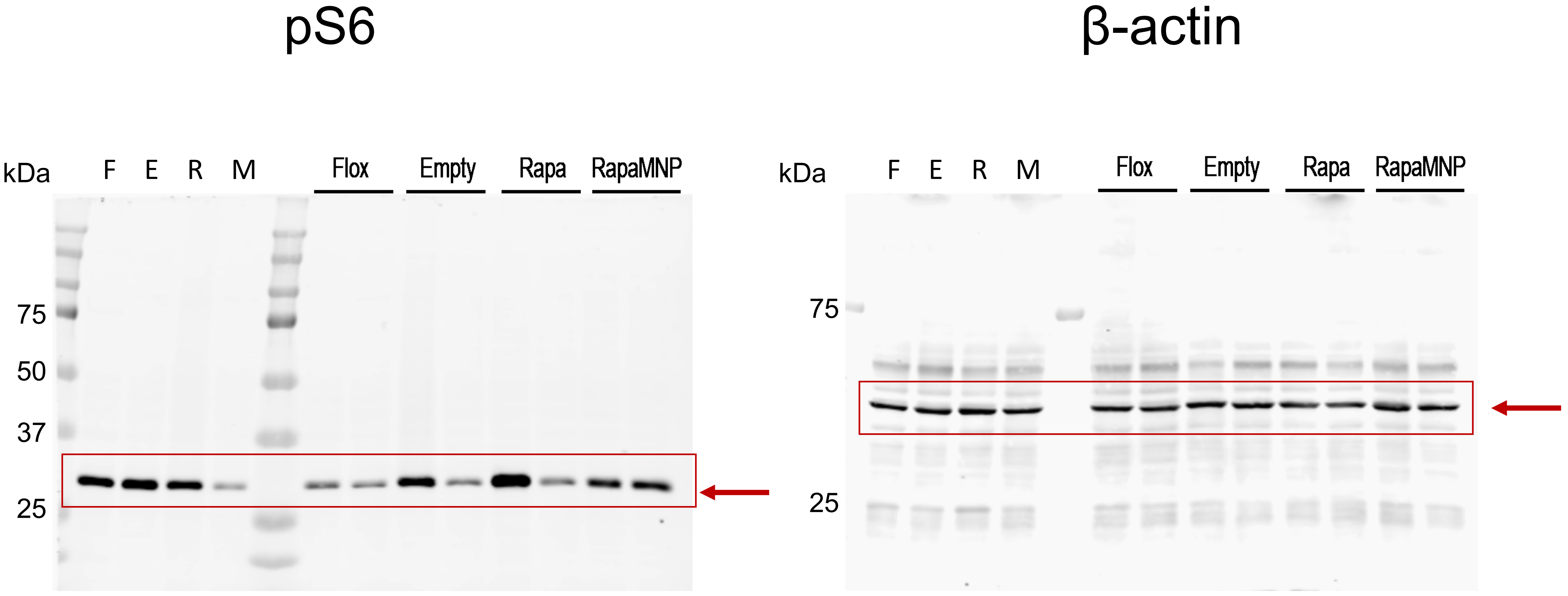

F- Flox E- Empty R-Rapa M- RapaMNP
